# Supplementary material for: Structural basis for ligand recognition of the neuropeptide Y Y2 receptor
Source: Nat Commun. 2021 Feb 2;12:737. doi: 10.1038/s41467-021-21030-9 (PMC7854658; doi:10.1038/s41467-021-21030-9)
Supplement: Supplementary file 1 — Supplementary Information [file 41467_2021_21030_MOESM1_ESM.pdf]

Supplementary Information

for

Structural basis for ligand recognition of the neuropeptide Y Y_2 receptor

Tingting Tang, Christin Hartig, et al.

Supplementary Information

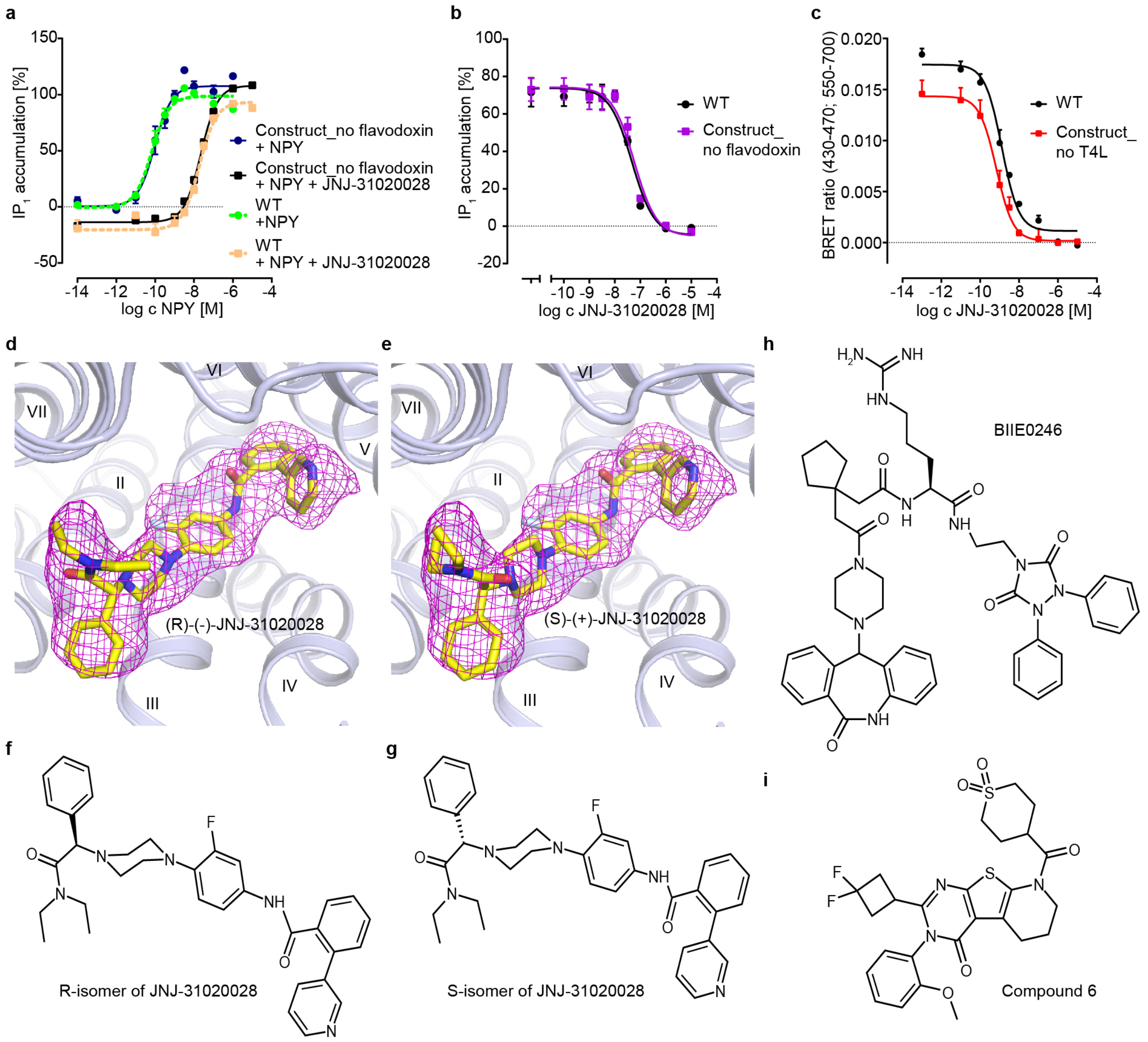

Supplementary Figure 1. Function validation of crystallization construct, electron densities of JNJ-31020028, and chemical structures of Y_2R antagonists. a, NPY-induced IP accumulation of Y_2R in the absence or presence of 1 μM JNJ-31020028. "Construct_no flavodoxin" indicates the modified Y_2R protein used for crystallization except that the ICL3-flavodoxin fusion was removed to allow G protein coupling. WT, wild-type Y_2R. Data are shown as mean ± SEM from at least four independent experiments performed in triplicate. See Supplementary Table 2 for detailed statistical evaluation. **b**, Inhibition of NPY-induced IP accumulation of Y_2R by JNJ-31020028. A fixed concentration of NPY (0.3 nM, ~ EC_{80}) was

used to stimulate IP accumulation. Data are shown as mean ± SEM from three independent experiments performed in triplicate (WT: IC_{50} = 4.8 nM, pIC_{50} ± SEM = 7.32 ± 0.10; construct_no flavodoxin: IC_{50} = 6.0 nM, pIC_{50} ± SEM = 7.22 ± 0.10). Data are normalized to the actual effective concentration (EC_x) at the day of the assay. **c**, NanoBRET-based binding assay of wild-type Y_2R (WT) and the modified Y_2R protein. "Construct_no T4L" indicates the modified Y_2R protein used for crystallization except that the N-terminal T4L fusion was removed to ensure suitable distance for BRET. Data are shown as mean ± SEM from three independent experiments performed in triplicate (WT: IC_{50} = 1.3 nM, pIC_{50} ± SEM = 8.89 ± 0.08; construct_no T4L: IC_{50} = 0.7 nM, pIC_{50} ± SEM = 9.17 ± 0.13). **d** and **e**, Electron densities of JNJ-31020028. **d**, R-isomer; **e**, S-isomer. Electron densities are contoured at 3.0 σ from a $|F_o|$-$|F_c|$ omit map and colored magenta. **f-i**, Chemical structures of representative antagonists of Y_2R. **f,** R-isomer of JNJ-31020028; **g**, S-isomer of JNJ-31020028; **h**, BIIE0246; **i**, Compound 6. Source data for panels a-c are provided as a Source Data file.

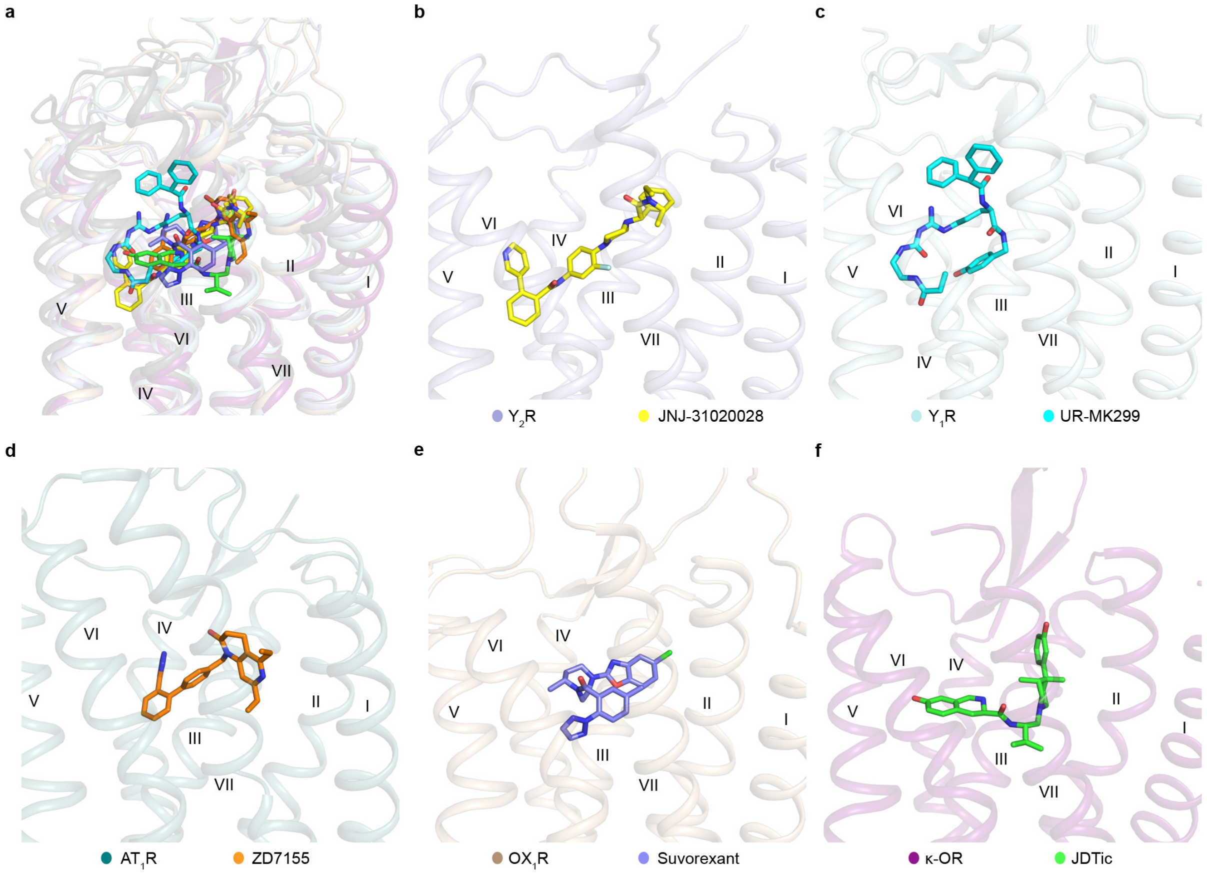

Supplementary Figure 2. Comparison of ligand-binding pockets in some class A peptide GPCR structures. a, Comparison of ligand-binding sites in Y_2R, Y_1R, AT_1R, OX_1R and κ-OR. The structures of Y_2R–JNJ-31020028, Y_1R–UR-MK299 (PDB code: 5ZBQ), AT_1R–ZD7155 (PDB code: 4YAY), OX_1R–suvorexant (PDB code: 4ZJ8) and κ-OR–JDTic (PDB code: 4DJH) are shown in cartoon representation and colored light blue, light cyan, green, gold, and purple, respectively. The ligands are shown as yellow, cyan, orange, blue, and green sticks, respectively. **b-f**, A close side view of the ligand in the binding pocket in each structure.

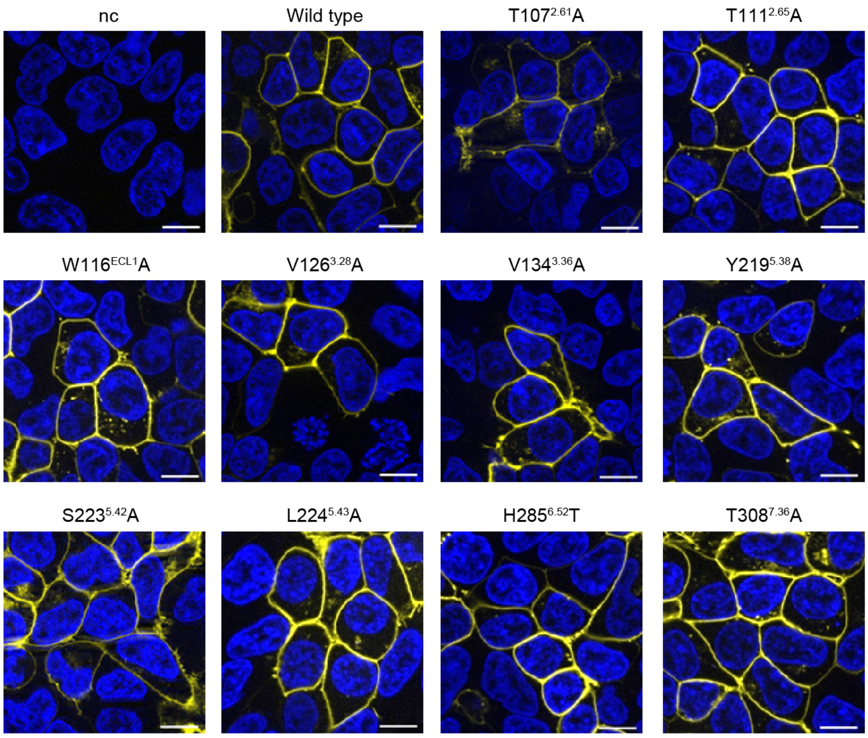

Supplementary Figure 3. Live-cell fluorescence microscopy of the wild-type Y_2R and several mutated receptor variants in transiently transfected HEK293 cells. All receptor variants were predominantly expressed in the cell membrane comparable to the wild-type receptor. The appropriate expression of the remaining mutants was already verified in previous studies[1,2,3]. Pictures are representative of two independent experiments with similar results. nc, negative control. Scale bars, 10 µm.

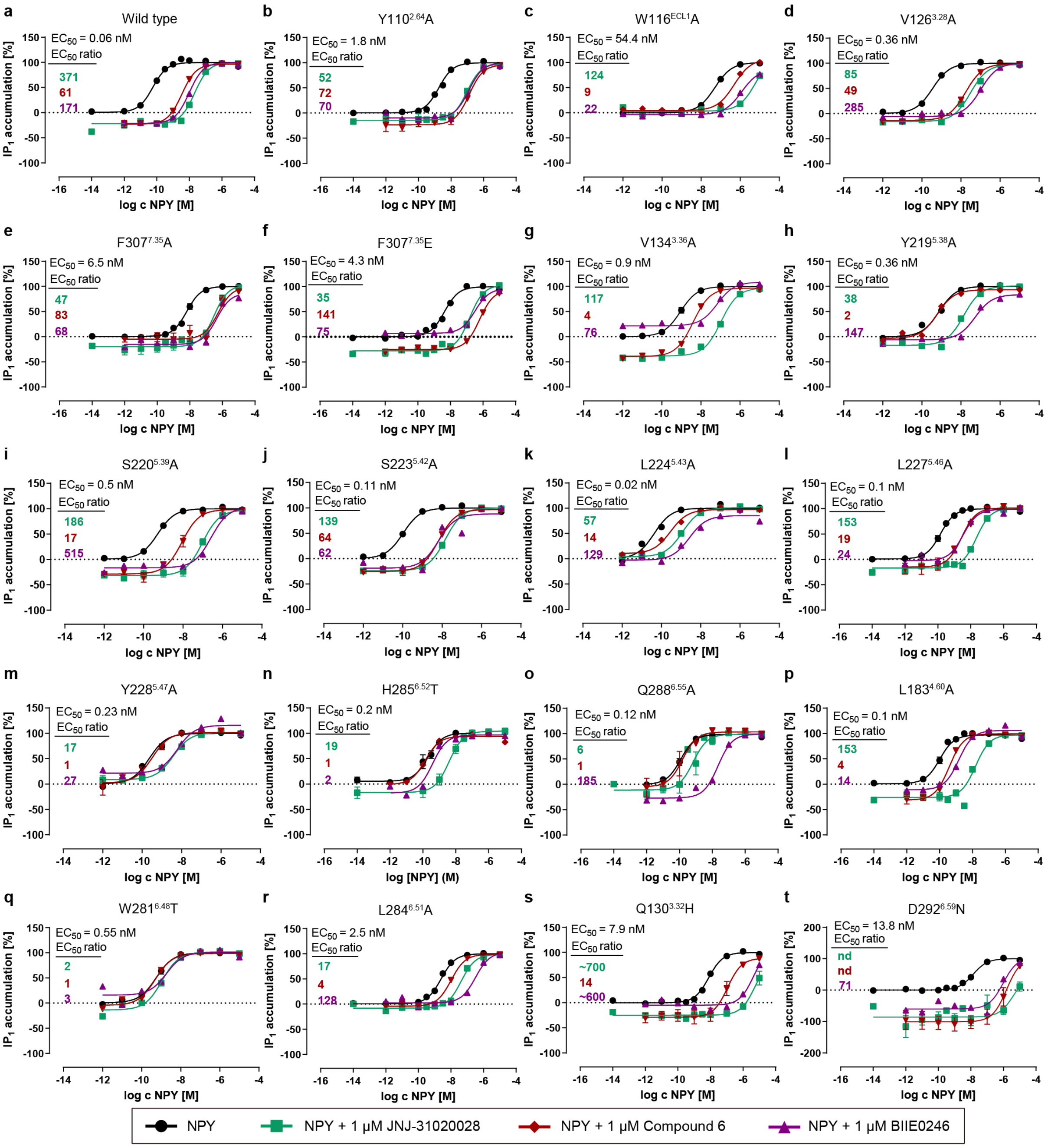

Supplementary Figure 4. IP accumulation assays. IP accumulation of wild-type and mutant Y_2 receptors induced by NPY (black) or NPY with the presence of antagonist JNJ-31020028 (1 µM, green), Compound 6 (1 µM, red) or BIIE0246 (1 µM, purple). At least two independent experiments were performed in triplicate. Where more than two experiments were performed, data are shown as mean ± SEM. Where two experiments were performed, data from a representative experiment are shown. See Supplementary Table 2 for detailed statistical

evaluation. **a**, Wild type; **b**, $Y110^{2.64}A$; **c**, $W116^{ECL1}A$; **d**, $V126^{3.28}A$; **e**, $F307^{7.35}A$; **f**, $F307^{7.35}E$; **g**, $V134^{3.36}A$; **h**, $Y219^{5.38}A$; **i**, $S220^{5.39}A$; **j**, $S223^{5.42}A$; **k**, $L224^{5.43}A$; **l**, $L227^{5.46}A$; **m**, $Y228^{5.47}A$; **n**, $H285^{6.52}T$; **o**, $Q288^{6.55}A$; **p**, $L183^{4.60}A$, **q**, $W281^{6.48}T$; **r**, $L284^{6.51}A$; **s**, $Q130^{3.32}H$; **t**, $D292^{6.59}N$. EC_{50} values of NPY (black) and EC_{50} ratios ($EC_{50\ (NPY+antagonist)}/EC_{50\ (NPY)}$) for antagonists (JNJ-31020028, green; Compound 6, red; BIIE0246, purple) are shown in the top left corner for each graph. A reduced EC_{50} ratio of the mutant compared to the wild-type receptor was interpreted as important for the respective antagonist. Source data are provided as a Source Data file.

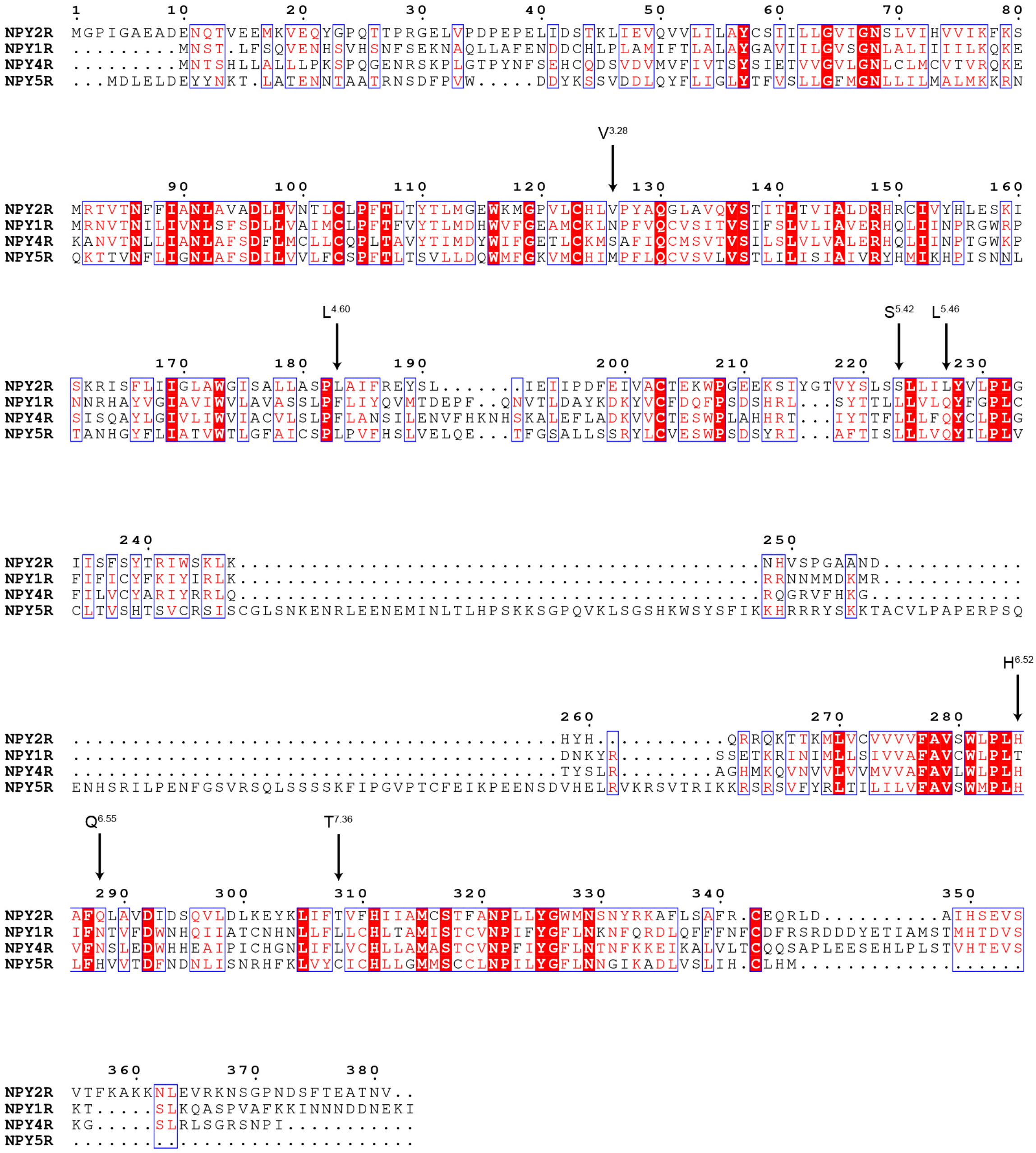

Supplementary Figure 5. Sequence alignment of the human NPY receptors. Sequence alignment of four human NPY receptors was performed by using align tools in UniProt database (http://www.uniprot.org/align/). The sequence conservation graph was prepared using the program ESPript 3.0 (http://espript.ibcp.fr/ESPript/cgi-bin/ESPript.cgi). The key Y_2R residues that are involved in JNJ-31020028 binding and variable in the NPY receptors are marked with black arrows.

Supplementary Table 1. Data collection and refinement statistics

	Y_2R–JNJ-31020028
**Data collection**[a]	
Space group	*C2*
Cell dimensions	
a, *b*, *c* (Å)	96.4, 50.9, 184.6
α, *β*, *γ* (°)	90.0, 90.7, 90.0
Resolution (Å)	50.0-2.80 (2.90-2.80)[b]
R_{pim} (%)	11.2 (51.7)
$I / \sigma(I)$	12.1 (1.6)
Completeness (%)	98.4 (97.2)
Redundancy	5.4 (4.1)
**Refinement**	
Resolution (Å)	37.7-2.80
No. reflections	21,815 (1,878)
R_{work} / R_{free} (%)	25.5 / 28.9
No. atoms	
Protein	3,415
Ligand	42
B-factors (Å^2)	
Protein	71.1
Ligand	70.3
R.m.s. deviations	
Bond lengths (Å)	0.011
Bond angles (°)	1.212
Ramachandran plot (%)	
Favored	96.3
Allowed	3.7
Disallowed	0.0

[a]Diffraction data from 52 crystals of Y_2R–JNJ-31020028 were used to solve the structure.
[b]Numbers in parentheses refer to the highest-resolution shell.

Supplementary Table 2. IP accumulation of wild-type (WT) and mutant Y_2 receptors for NPY and antagonists.

Mutants	NPY		NPY/JNJ-31020028 (1 μM)				NPY/Compound 6 (1 μM)				NPY/BIIE0246 (1 μM)			
	EC_{50} (nM) (pEC_{50} ± SEM)[a]	n[b]	EC_{50} (nM) (pEC_{50} ± SEM)	Ratio[c]	Ratio change (fold)[d]	n	EC_{50} (nM) (pEC_{50} ± SEM)	Ratio	Ratio change (fold)	n	EC_{50} (nM) (pEC_{50} ± SEM)	Ratio	Ratio change (fold)	n
WT	0.06 (10.24 ± 0.04)	13	21 (7.67 ± 0.06)	371	1	8	3.5 (8.46 ± 0.07)	61	1	8	9.1 (8.2; 9.8)	159 (144; 171)	1	2
Construct[e]	0.09 (10.02 ± 0.10)	5	23 (7.65 ± 0.14)	237	1.6	4	/	/	/	/	/	/	/	/
Y110$^{2.64}$A	1.8 (8.75 ± 0.04)	9	94 (7.03 ± 0.09)	52	7	3	129 (6.89 ± 0.11)	72	0.8	3	45; 126	25; 70	6; 2	2
T111$^{2.65}$A	2.0 (1.3; 2.9)	2	513; 1,715	257; 860	1.4; 0.4	2	89; 82	45; 41	1.4; 1.5	2	530; 459	265; 230	0.6; 0.7	2
W116^{ECL1}A	54 (58; 51)	2	6,727; 4,442	124; 82	3; 5	2	490; 330	9; 6	7; 10	2	1,180; 2,974	22; 55	7; 3	2
V126$^{3.28}$A	0.36 (9.45 ± 0.06)	4	36; 30	100; 85	4; 4	2	18; 12	49; 34	1.2; 1.8	2	102; 114	285; 320	0.6; 0.5	2
V126$^{3.28}$N	5.5 (8.26 ± 0.12)	4	570 (6.24 ± 0.15)	104	4	4	/	/	/	/	/	/	/	/
Q130$^{3.32}$H	7.9 (8.10 ± 0.05)	8	nd	nd	nd	3	113 (6.95 ± 0.16)	14	4	3	nd	nd	nd	2
V134$^{3.36}$A	0.90 (9.04 ± 0.05)	4	106; 73	117; 80	3; 5	2	4.0; 4.4	4; 5	15; 12	2	69; 79	76; 87	2; 1.8	2
L183$^{4.60}$A	0.10 (9.99 ± 0.07)	8	16 (7.81 ± 0.10)	153	2	3	0.40 (9.41 ± 0.07)	3.8	16	3	0.65; 1.5	6; 14	27; 11	2
L183$^{4.60}$F	0.23 (9.64 ± 0.14)	4	124 (6.91 ± 0.11)	535	0.7	4	/	/	/	/	/	/	/	/
Y219$^{5.38}$A	0.36 (0.76; 0.23)	2	14; 18	38; 50	10; 7	2	0.99; 0.62	3; 2	20; 31	2	18; 53	49; 147	3; 1	2
S220$^{5.39}$A	0.52 (9.29 ± 0.06)	6	97 (7.02 ± 0.09)	186	2	4	9.0 (8.05 ± 0.09)	17	4	4	267; 254	515; 490	0.3; 0.3	2
S223$^{5.42}$A	0.11 (9.98 ± 0.05)	6	14 (7.83 ± 0.05)	139	3	4	6.7 (8.17 ± 0.07)	64	1	4	29; 6.5	276; 62	0.6; 3	2
S223$^{5.42}$L	0.23 (9.64 ± 0.10)	4	197 (6.71 ± 0.13)	865	0.4	4	/	/	/	/	/	/	/	/
L224$^{5.43}$A	0.02 (0.04; 0.01)	2	1.2; 2.6	57; 126	7; 3	2	0.29; 0.49	14; 24	4; 3	2	28; 2.6	1,349; 129	0.1; 1.2	2
L227$^{5.46}$A	0.14 (9.84 ± 0.05)	9	22 (7.65 ± 0.07)	153	2	5	2.8 (8.55 ± 0.13)	19	3	3	4.6; 3.5	31; 24	5; 7	2
L227$^{5.46}$Q	0.14 (9.86 ± 0.15)	4	6.9 (8.16 ± 0.14)	50	7	4	/	/	/	/	/	/	/	/
Y228$^{5.47}$A	0.23 (9.64 ± 0.07)	5	3.8 (8.42 ± 0.09)	17	22	3	0.32 (9.49 ± 0.14)	1.4	44	3	2.4; 6.2	10; 27	16; 6	2
W281$^{6.48}$T	0.55 (9.26 ± 0.07)	5	1.1 (8.96 ± 0.07)	2	186	3	0.50 (9.30 ± 0.08)	0.9	68	3	1.8; 1.1	3; 2	48; 80	2
L284$^{6.51}$A	2.5 (8.60 ± 0.03)	8	42 (7.37 ± 0.06)	17	22	3	10 (7.99 ± 0.05)	4	15	3	223; 322	89; 128	1.8; 1.2	2
H285$^{6.52}$T	0.20 (9.69 ± 0.10)	3	3.9 (8.41 ± 0.13)	19	20	3	0.26; 0.09	1.3; 0.4	47; 153	2	0.72; 0.38	4; 1.9	45; 84	2
Q288$^{6.55}$A	0.12 (9.92 ± 0.07)	8	0.68 (9.17 ± 0.18)	6	65	3	0.16 (9.81 ± 0.19)	1.3	47	3	12; 22	102; 185	1.6; 0.9	2
Q288$^{6.55}$N	0.14 (9.86 ± 0.11)	4	0.29 (9.54 ± 0.13)	2	186	4	/	/	/	/	/	/	/	/
D292$^{6.59}$N	14 (7.86 ± 0.05)	8	nd	nd	nd	3	nd	nd	nd	3	970; 865	71; 63	2; 3	2
F307$^{7.35}$A	6.5 (8.19 ± 0.05)	8	305 (6.52 ± 0.13)	47	8	3	540 (6.27 ± 0.23)	83	0.7	3	443; 723	68; 111	2; 1.4	2
F307$^{7.35}$E	4.3 (8.36 ± 0.09)	9	151 (6.82 ± 0.07)	35	11	3	655; 615	151; 141	0.4; 0.4	2	327; 1,693	75; 389	2; 0.4	2
T308$^{7.36}$A	0.25 (0.34; 0.20)	2	171; 161	695; 655	0.5; 0.6	2	24; 28	99; 112	0.6; 0.5	2	61; 54	248; 219	0.6; 0.7	2
T308$^{7.36}$L	0.12 (9.92 ± 0.14)	4	54 (7.27 ± 0.12)	454	0.8	4	/	/	/	/	/	/	/	/

[a]Data are shown as mean ± SEM from at least three independent experiments or the results of two individual experiments each performed in triplicate. nd, not determined. /, not tested.
[b]Sample size; the number of independent experiments performed in triplicate.

[c]The EC_{50} ratio refers to the shift between the NPY and NPY + 1 μM antagonist curve ($EC_{50(NPY + antagonist)}/EC_{50(NPY)}$) and characterizes the antagonistic effect on the wild-type receptor or receptor mutants. By comparison of EC_{50} ratios between wild-type and mutant receptors, influences of all tested residues on antagonistic activity were determined. A higher ratio indicates higher antagonistic activity. A reduced EC_{50} ratio of mutant compared to the wild-type receptor was interpreted as important for the respective antagonist.
[d]Fold change of EC_{50} ratio refers to EC_{50} ratio (WT)/EC_{50} ratio (mutant).
[e]"Construct" indicates the modified Y_2R protein used for crystallization except that the ICL3-flavodoxin fusion was removed to allow G protein coupling.
Source data are provided as a Source Data file.

Supplementary Table 3. Primers used in this work.

	Primer sequences (5'-3')
Y_2R-WT-F	ATTGGCGCGCCGATGGGACCAATCGGAGCAGAAGCA
T4L-F	ATTGGCGCGCCGAACATCTTCGAGATGCTGCGTATC
Y_2R-T4L-R	TGCTCCGATTGGTCCCATGTAAGCGTCCCATGTACC
T4L-Y_2R-F	GGTACATGGGACGCTTACATGGGACCAATCGGAGCA
Flavodoxin-F	AAGCTGAAAAACCACGTCGCCAAGGCTCTCATCGTG
Flavodoxin-R	GCGCTGATGGTAGTGGTCAATAGCGCCCCTCACGTC
Flavodoxin-Y_2R-F	GACGTGAGGGGCGCTATTGACCACTACCATCAGCGC
Flavodoxin-Y_2R-R	CACGATGAGAGCCTTGGCGACGTGGTTTTTCAGCTT
C-truncation-R	ATTGAATTCGACTTCGGAGTGGATTGCGTCCAAACG
Y110A-F	CTCTTACCgctACCTTAATGGGGGAGTGGAAA
Y110A-R	TAAGGTagcGGTAAGAGTGAACGGTAGACACAGAG
T111A-F	ACCTATgccTTAATGGGGGAGTGGAAAATGGG
T111A-R	CCCATTAAggcATAGGTAAGAGTGAACGGTAGACACAG
W116A-F	ATGGGGGAGgcgAAAATGGGTCCTGTCCTGTGC
W116A-R	ATTTTcgcCTCCCCCATTAAGGTATAGGTAAGAG
V126A-F	CCACCTGgcgCCCTATGCCCAGGGCCTGGCAG
V126A-R	CATAGGGcgcCAGGTGGCACAGGACAGGACCC
V126N-F	CCACCTGaacCCCTATGCCCAGGGCCTGGCAG
V126N-R	CATAGGGgttCAGGTGGCACAGGACAGGACCC
Q130H-F	TGGTGCCCTATGCCcacGGCCTGGCAGTACAAGTATCCA
Q130H-R	gtgGGCATAGGGCACCAGGTGGCACAGGACAG
V134A-F	CCTGGCAgcaCAAGTATCCACAATCACCTTGACAG
V134A-R	ATACTTGtgcTGCCAGGCCCTGGGCATAGGGC
L183A-F	AAGTCCCgcgGCCATCTTCCGGGAGTATTCGC
L183A-R	AGATGGCcgcGGGACTTGCCAGCAGGGCACTG
L183F-F	AAGTCCCttcGCCATCTTCCGGGAGTATTCGC
L183F-R	AGATGGCgaaGGGACTTGCCAGCAGGGCACTG
Y219A-F	CACTGTCgctAGTCTTTCTTCCTTGTTGATCTTGTATG
Y219A-R	AAAGACTagcGACAGTGCCATAGATGCTCTTCTC
S220A-F	GCACTGTCTATgctCTTTCTTCCTTGTTGATCTTGTATGTT
S220A-R	AAGagcATAGACAGTGCCATAGATGCTCTTCT
S223A-F	GTCTTTCTgccTTGTTGATCTTGTATGTTTTGCCTC
S223A-R	CAACAAggcAGAAAGACTATAGACAGTGCCATAGATGC
S223L-F	GTCTTTCTctcTTGTTGATCTTGTATGTTTTGCCTC
S223L-R	CAACAAgagAGAAAGACTATAGACAGTGCCATAGATGC
L224A-F	TTCTTCCgcgTTGATCTTGTATGTTTTGCCTCTGG
L224A-R	AGATCAAcgcGGAAGAAAGACTATAGACAGTGCCATA
L227A-F	CTTGTTGATCgcgTATGTTTTGCCTCTGGGCATTA

L227A-R	CATAcgcGATCAACAAGGAAGAAAGACTATAGACAG
L227Q-F	CcagTATGTTTTGCCTCTGGGCATTATATCAT
L227Q-R	GAGGCAAAACATActgGATCAACAAGGAAGAAAGACTATAGACAG
Y228A-F	GATCTTGgctGTTTTGCCTCTGGGCATTATATC
Y228A-R	GCAAAACagcCAAGATCAACAAGGAAGAAAGACTATAGA
W281T-F	TTTGCGGTCAGCacgCTGCCTCTCCATGCCTTCC
W281T-R	AGcgtGCTGACCGCAAACACCACCACCACACA
L284A-F	CTGCCTgccCATGCCTTCCAGCTTGCCGTTGA
L284A-R	AAGGCATGggcAGGCAGCCAGCTGACCGCAAA
H285T-F	TCactGCCTTCCAGCTTGCCGTTGACATTGAC
H285T-R	AAGCTGGAAGGCagtGAGAGGCAGCCAGCTGACC
Q288A-F	TCCATGCCTTCgcgCTTGCCGTTGACATTGACAGC
Q288A-R	AAGcgcGAAGGCATGGAGAGGCAGCCAGCTGA
Q288N-F	ATGCCTTCaacCTTGCCGTTGACATTGACAGC
Q288N-R	GGCAAGgttGAAGGCATGGAGAGGCAGCCAGC
D292N-F	GCCGTTaacATTGACAGCCAGGTCCTGGACCT
D292N-R	CTGTCAATgttAACGGCAAGCTGGAAGGCATG
F307A-F	ACTCATCgccACAGTGTTCCACATCATCGCCA
F307A-R	ACACTGTggcGATGAGTTTGTACTCCTTCAGGTCC
F307E-F	ACTCATCgagACAGTGTTCCACATCATCGCCA
F307E-R	ACACTGTctcGATGAGTTTGTACTCCTTCAGGTCC
T308A-F	CATCTTCgcaGTGTTCCACATCATCGCCATGT
T308A-R	GGAACACtgcGAAGATGAGTTTGTACTCCTTCAGGTC
T308L-F	CATCTTCctaGTGTTCCACATCATCGCCATGT
T308L-R	GGAACACtagGAAGATGAGTTTGTACTCCTTCAGGTC

Supplementary references

1. Burkert, K. et al. A deep hydrophobic binding cavity is the main interaction for different Y_2R antagonists. *ChemMedChem* **12**, 75-85 (2017).
2. Kaiser, A. et al. G protein preassembly rescues efficacy of $W^{6.48}$ toggle mutations in neuropeptide Y_2 receptor. *Mol. Pharmacol.* **93**, 387-401 (2018).
3. Kaiser, A. et al. Unwinding of the C-terminal residues of neuropeptide Y is critical for Y_2 receptor binding and activation. *Angew. Chem.* **54**, 7446-7449 (2015).
